# Inferring Phytoplankton, Terrestrial Plant and Bacteria Bulk δ¹³C Values from Compound Specific Analyses of Lipids and Fatty Acids

**DOI:** 10.1371/journal.pone.0133974

**Published:** 2015-07-24

**Authors:** Sami J. Taipale, Elina Peltomaa, Minna Hiltunen, Roger I. Jones, Martin W. Hahn, Christina Biasi, Michael T. Brett

**Affiliations:** 1 Lammi Biological Station, University of Helsinki, Lammi, Finland; 2 Department of Biology, University of Eastern Finland, Joensuu, Finland; 3 Department of Biological and Environmental Science, University of Jyväskylä, Jyväskylä, Finland; 4 Research Institute for Limnology, University of Innsbruck, Mondsee, Austria; 5 Department of Environmental Sciences, University of Eastern Finland, Kuopio, Finland; 6 Department of Civil and Environmental Engineering, University of Washington, Seattle, Washington, United States of America; National Taiwan University, TAIWAN

## Abstract

Stable isotope mixing models in aquatic ecology require δ^13^C values for food web end members such as phytoplankton and bacteria, however it is rarely possible to measure these directly. Hence there is a critical need for improved methods for estimating the δ^13^C ratios of phytoplankton, bacteria and terrestrial detritus from within mixed seston. We determined the δ^13^C values of lipids, phospholipids and biomarker fatty acids and used these to calculate isotopic differences compared to the whole-cell δ^13^C values for eight phytoplankton classes, five bacterial taxa, and three types of terrestrial organic matter (two trees and one grass). The lipid content was higher amongst the phytoplankton (9.5±4.0%) than bacteria (7.3±0.8%) or terrestrial matter (3.9±1.7%). Our measurements revealed that the δ^13^C values of lipids followed phylogenetic classification among phytoplankton (78.2% of variance was explained by class), bacteria and terrestrial matter, and there was a strong correlation between the δ^13^C values of total lipids, phospholipids and individual fatty acids. Amongst the phytoplankton, the isotopic difference between biomarker fatty acids and bulk biomass averaged -10.7±1.1‰ for Chlorophyceae and Cyanophyceae, and -6.1±1.7‰ for Cryptophyceae, Chrysophyceae and Diatomophyceae. For heterotrophic bacteria and for type I and type II methane-oxidizing bacteria our results showed a -1.3±1.3‰, -8.0±4.4‰, and -3.4±1.4‰ δ^13^C difference, respectively, between biomarker fatty acids and bulk biomass. For terrestrial matter the isotopic difference averaged -6.6±1.2‰. Based on these results, the δ^13^C values of total lipids and biomarker fatty acids can be used to determine the δ^13^C values of bulk phytoplankton, bacteria or terrestrial matter with ± 1.4‰ uncertainty (i.e., the pooled SD of the isotopic difference for all samples). We conclude that when compound-specific stable isotope analyses become more widely available, the determination of δ^13^C values for selected biomarker fatty acids coupled with established isotopic differences, offers a promising way to determine taxa-specific bulk δ^13^C values for the phytoplankton, bacteria, and terrestrial detritus embedded within mixed seston.

## Introduction

Stable isotope analyses (SIA) are increasingly used to investigate food web structure and carbon transfer pathways in aquatic ecosystems. For example, SIA have been pivotal in developing ideas about the relative contributions of autochthonous and allochthonous carbon sources in lake food webs [1–6]. Application of SIA has also underpinned much of the work that has revised views on the importance of littoral and benthic processes in lake ecosystems [7]. These approaches generally use isotope mixing models (e.g. IsoSource, SIAR) [8, 9] to analyze the data, and these mixing models require carbon isotope values (δ^13^C) for various end members, often including phytoplankton, bacteria and terrestrial organic matter. Whereas the δ^13^C of diets can be easily measured in laboratory experiments [10], it is rarely possible to physically separate phytoplankton, bacteria, or terrestrial detritus from other components of the seston in order to determine their δ^13^C values directly. Grey et al. [1] separated large diatoms from Loch Ness seston samples by repeated sedimentation and determined their δ^13^C, while Vuorio et al. [11] measured δ^13^C directly from various phytoplankton taxa from Finnish lakes after manual separation of colonies under microscopy, but such physical separation is rarely feasible and the high time demands for such methods make them impractical for routine use. Furthermore, the approach applied by these authors can only be applied to large or colonial phytoplankton taxa which may be also too large for most zooplankton to ingest. Additionally, there are large differences in diet quality amongst phytoplankton classes for zooplankton; for example, copepods feed selectively on high quality phytoplankton and do not consume all phytoplankton taxa evenly [12–14]. Vuorio et al. [11] also showed there can be large differences in stable isotopes values between the main phytoplankton taxa. Hence obtaining robust δ^13^C values for phytoplankton and bacteria has proven to be an enduring problem for the SIA approach to aquatic food web studies and often results in high uncertainties for diet contributions to zooplankton [15].

Because direct determinations are usually not feasible, researchers have resorted to a variety of indirect approaches. One approach has been to use values from appropriate primary consumers as a surrogate for primary producer values (e.g. unionid mussel values to represent phytoplankton; [16]). Although this approach has the advantage of time-integration it suffers from considerable uncertainty and of course also lacks the information about specific primary producer δ^13^C values that would be very valuable in many contexts. This approach also assumes the analyst actually knows the true diet of the primary consumer, which is never actually true in natural systems. Marty & Planas [17] reviewed the indirect approaches that have been used to estimate actual phytoplankton δ^13^C values. These include: 1) determining the δ^13^C value of dissolved inorganic carbon (DIC) and applying an assumed fractionation factor between DIC and phytoplankton; 2) simply using the δ^13^C of POM as an estimate of the phytoplankton signature; 3) correcting the δ^13^C of POM according to estimates of its phytoplankton proportion; and 4) ^13^C-enriching phytoplankton to obtain a clearer separation from bacteria or allochthonous POM and then applying methods 1–3. All of these approaches have obvious weaknesses and none is entirely satisfactory. The most widely used approach is probably 1), but not all studies are able to determine δ^13^C of DIC empirically, and as values differ widely between lakes [18] and with season [19] using literature values is not appropriate [20]. Moreover, the carbon isotope fractionation between DIC and phytoplankton is highly variable [18, 19] so that again assuming a value based on literature reports is fraught with uncertainty [15]. In the case of bacteria even less is known. Heterotrophic bacteria are generally assumed to have similar δ^13^C signatures to their organic matter source (for example allochthonous dissolved organic matter, DOM), and there is some justification for this assumption [21]. The δ^13^C of methane-oxidizing bacteria or photoautotrophic green sulphur bacteria has usually been estimated from the corresponding values for the carbon substrate (CH_4_ or DIC) and using fractionation factors obtained from the literature. However, these fractionation factors have a very wide reported range, e.g., -7.8 to -28.4‰ for methane oxidizing bacteria (MOB) and -12.0 to -13.7‰ for photoautotrophic bacteria [22, 23, 24].

An alternative approach is to extract certain biochemical components associated with particular organisms from seston samples and determining the δ^13^C values of these biochemicals directly. Provided the relationship between these values and those of the bulk organism biomass are known and well constrained, the δ^13^C values of the bulk matter can then be estimated robustly. Algal pigments and fatty acids (FAs) have been used as chemotaxonomic markers for freshwater and marine phytoplankton because these biomolecules vary greatly amongst phytoplankton classes [10, 25, 26]. Analyses of the δ^13^C values of chlorophyll-a have been used to obtain a δ^13^C value for phytoplankton in mixed seston [27, 28], and the carbon isotope differences between chlorophyll and whole algal cells appears to be rather constant [28]. However, the chlorophyll approach cannot distinguish different phytoplankton taxa, which is often important for consumers like zooplankton (e.g., *Eudiaptomus*) which are known to feed selectively [12]. Potentially more useful would be compound-specific carbon isotope analyses of the δ^13^C values of molecules such as FAs, and especially phospholipid fatty acids (PLFAs) [29, 30], several of which can be used as diagnostic biomarkers for specific algae and bacteria taxa. Carbon isotopes of FA biomarkers have been used in a variety of ecological studies [20, 30, 31, 32, 33, 34, 35]. However, the δ^13^C PLFA approach could be used even more effectively for δ^13^C determination of distinct bacteria, phytoplankton and terrestrial plants if data for δ^13^C difference between individual PLFA and bulk cell material of bacteria or phytoplankton were better known. At present such differences have only been reported for saturated FAs [36, 37] and not from specific FA biomarkers, and only for a few phytoplankton taxa.

The δ^13^C of bulk biomass is the sum of different carbon containing organic molecules [36]:

$$\delta^{13}C_{biomass}=X_{CNA}\delta^{13}C_{NA}+X_{C\mathrm{Pr}ot}\delta^{13}C_{\mathrm{Pr}ot}+X_{CSacc}\delta^{13}C_{Sacc}+X_{CLip}\delta^{13}C_{Lip}$$ (1)

where X_C_ is mole fraction of carbon and subscripts NA, Prot, Sacc and Lip refer to nucleic acids, proteins, saccharides and lipids, respectively. Autotrophs synthesize these carbon containing biomolecules from CO_2_ via different enzymatic pathways which generates δ ^13^C differences between bulk tissue and biomolecules [36, 38].

In order to use cellular fatty acids for δ^13^C determination of bulk tissue, we studied the integrity and stability of the δ ^13^C values of lipids amongst different freshwater algae and bacteria under culture conditions similar to those commonly found in boreal lakes in the summer. Here we present new data for δ^13^C differences between bulk cell biomass and total lipids, phospholipids and FAs for 22 phytoplankton strains (including 4 cyanobacteria), 5 non-phototrophic bacterial species, and 3 terrestrial plants. We also discuss whether and how lipids and FAs can be used as a tool to infer the bulk δ^13^C values of phytoplankton, bacteria and terrestrial matter.

## Materials and Methods

### Phytoplankton and bacteria culturing

We cultured phytoplankton and bacteria representing a wide range of phylogenetic and functional groups. Table 1 lists the different strains cultured with information about their source, and the culture media used. Some strains were cultured independently during different years to evaluate the repeatability of the δ^13^C difference within a taxon under similar conditions. The selected phytoplankton represent eukaryotic algae and cyanobacteria typical of freshwater lakes. The strains were grown either at 20°C or at 18°C with a light:dark-cycle of 16L:8D or 14L:10D and in growth media specific for the strains (Table 1). We used plastic or glass vials with volume > 200 mL. Depending on the cell density, 0.5–3 mL of the phytoplankton stock was inoculated per 100 mL of fresh culture media every two weeks. The condition of the cells was examined under a microscope and samples for phytoplankton analysis were harvested in the late phase of exponential growth, usually 2–3 weeks after the inoculation.

**Table 1:** Culture conditions used for growth of phytoplankton, and bacteria, and details of terrestrial material analysed. Phytoplankton and bacteria strains used for this study were obtained from different culture collections and universities. They were cultured using optimal media for each strain. Phytoplankton cultures were maintained under either a 14:10 or 16:8 h light:dark cycle. Temperature of all phytoplankton cultures was 18−20°C (average±SD) and bacteria 23−30°C (average±SD). Terrestrial matter includes finely ground particles of leaves from one grass (*Phragmites*) and two tree (*Betula* and *Alnus*) taxa. Due to the high number of strains we were not able to perform all analysis from all cultured strains. UWCC: Algal and Fungal Culture Collection of University of Washington, Seattle, Washington, USA; Peltomaa: Lammi Biological Station, University of Helsinki, Finland; CCAP: Culture Collection of Algae and Protozoa, Ambleside, Cumbria, UK; Gilbert: Dartmouth College, New Hampshire, USA; NIVA: Norwegian Institute for Water Research, Oslo, Norway; UTEX: University of Texas Culture Collection, University of Texas at Austin, Texas, USA; CPCC: Canadian Phycological Culture Centre, University of Waterloo, Ontario, Canada; CCMP: National Center for Marine Algae and Microbiota, Bigelow Laboratory for Ocean Sciences, Maine, USA; Carolina: Carolina Biological Supply Company, Burlington, North Carolina, USA. CFA = compound-specific SIA, TLS = lipid stable isotope, PLS = phospholipid stable isotope, C% = carbon content (%), TL% = lipid content (%) and PL% = phospholipid content (%).

Species	Strain number	Collection	Place Cultured	Media	Light cycle	Temperature (°C)	Analysis
**Phytoplankton**							
Chlorophyceae (Green algae)							
*Chlamydomonas reinhardtii*	1	UWCC^1^	Universtity of Washington	L16 (Lindström 1983)	14:10	18±1	CFA, C%
*Selenastrum capricornutum*	2	UWCC^1^	Universtity of Washington	L16 (Lindström 1983)	14:10	18±1	CFA, C%
*Scenedesmus gracilis*	3	University of Basel	Universtity of Jyväskylä	WC (Guillard and Lorenzen 1972, Guillard 1975)	14:10	20±1	CFA, PLS, C%, PL%
*Monoraphidium griffithii*	4	NIVA-CHL8	Universtity of Jyväskylä	WC (Guillard and Lorenzen 1972, Guillard 1975)	14:10	20±1	PLS, C%, PL%
Euglenophyceae (Euglenoids)							
*Euglena gracilis*	5	CCAP^3^ 1224/5Z	University of Helsinki		16:8	20±1	CFA, C%
Chrysophyceae (Golden algae)							
*Mallomonas caudata*	6	CCAP^3^ 929/8	University of Helsinki	WC (Guillard and Lorenzen 1972, Guillard 1975)	16:8	20±1	CFA, TLS, C%, TL%
*Synura sp*.	7	SCCAP K-1875	University of Helsinki	WC (Guillard and Lorenzen 1972, Guillard 1975)	16:8	20±1	CFA, C%
Raphidophyceae (Raphidophyte algae)							
*Gonyostomum semen*	8	GSB 02**/04***	University of Washington	L16 (Lindström 1983)	14:10	18±1	CFA, C%
Cryptophyceae (Cryptomonads)							
*Cryptomonas sp*.	9	SCCAP K-1876	University of Helsinki	AF6 (Watanabe et al. 2000)	16:8	20±1	CFA, C%
*Cryptomonas erosa*	10	Gilbert^4^, U.S.A.*	Universtity of Ottago		16:8	20±1	TLS, PLS, C%, TL%, PL%
*Crytomonas ozolinii*	11	UTEX^6^ LB 2782	University of Washington	L16 (Lindström 1983)	14:10	18±1	CFA, TLS, C%, TL%
*Rhodomonas minuta*	12	CPCC^7^ 344	University of Washington	L16 (Lindström 1983)	14:10	18±1	CFA
*Rhodomonas lacustris*	13	NIVA^5^ 8/82	University of Washington	L16 (Lindström 1983)	14:10	18±1	TLS, PLS, C%, TL%, PL%
Bacillariophyceae (Diatoms)							
*Fragilaria crotonensis*	14	UTEX^6^ LB FD56	Universtity of Washington	Diatom medium (Beakes et al. 1986)	14:10	18±1	CFA, C%
*Cyclotella meneghiniana*	15	PAE Lab, Belgium	Universtity of Washington	Diatom medium (Beakes et al. 1986)	14:10	18±1	CFA, C%
*Navicula pellicosa*	16	UTEX^6^ B664	Universtity of Washington	Diatom medium (Beakes et al. 1986)	14:10	18±1	TLS, PLS, C%, TL%, PL%
*Diatoma tenuis*	17	CPCC 62	University of Jyväskylä	Chu-10	14:10	20±1	TLS, C%, TL%
Dinophyceae (Dinoflagellates)							
*Peridinium cintum*	18	SCCAP K-1721	University of Jyväskylä	WC + Se (Guillard and Lorenzen 1972, Guillard 1975)	14:10	20±1	TLS, PLS, C%, TL%, PL%
Cyanophyceae (Cyanobacteria)							
*Synechococcus elongatus*	19	UTEX LB 563	University of Washington	L16 (Lindström 1983)	14:10	18±1	CFA,TLS, PLS, C%, TL%, PL%
*Microcystis aeruginosa*	20	UTEX LB 2063	University of Washington	L16 (Lindström 1983)	14:10	18±1	CFA,
*Limnothrix planctonica*	21	NIVA-CYA 107	Universtity of Jyväskylä	WC (Guillard and Lorenzen 1972, Guillard 1975)	14:10	20±1	TLS, PLS, C%, TL%, PL%
*Pseudanabaena limnetica*	22	NIVA 276/11	Universtity of Jyväskylä	WC (Guillard and Lorenzen 1972, Guillard 1975)	14:10	20±1	TLS, PLS, C%, TL%, PL%
**Terrestrial matter**							
*Pragmites australis*	23		University of Eastern Finland				CFA,TLS, PLS, C%, TL%, PL%
*Alnus rubra*	24		University of Washington				CFA,TLS, PLS, C%, TL%, PL%
*Betula nana*	25		University of Jyväskylä				CFA,TLS, PLS, C%, TL%, PL%
**Heterotrophic bacteria (Gram+, Actinobacteria)**							
Actinobacterium *Candidatus* Rhodoluna limnophila	26	MWH-VicMua1	University of Innsbruck	NSY medium (Hahn et al. 2004)		24±1	CFA,TLS, PLS, C%, TL%, PL%
**Heterotrophic bacteria (Gram-, Proteobacteria, Betaproteobacteria)**							
Betaproteobacterium *Polynucleobacter necessarius* ssp. *asymbioticus*	27	MWH-Mekk-D6	University of Innsbruck	NSY medium (Hahn et al. 2004)		24±1	CFA,TLS, PLS, C%, TL%, PL%
**Autotrophic green sulfur bacteria (Phylum Chlorobi)**							
*Chlorobium phaerobacteroidetes*	28	DSM 267	DSMZ				CFA, C%
**Methane oxidizing bacteria (Type I, Proteobacteria, Gammaproteobacteria)**							
*Methylobacter tundripaludum*	29	SV96^T^	University of Jyväskylä	M2 medium (DSMZ medium 921)		23±1	CFA, C%
*Methylomonas methanica*	30	LW13	University of Washington	NMS (Whittenbury, Philips & Wilkinson, 1970)		30±1	CFA, C%
**Methane oxidizing bacteria (Type II, Proteobacteria, Gammaproteobacteria)**							
*Methylosinus trichosporium*	31	OB3b	University of Jyväskylä	NMS (Whittenbury, Philips & Wilkinson, 1970)		30±1	CFA, C%

The two heterotrophic bacterial strains studied are archetypal lake bacteria; Actinobacterium *Candidatus* Rhodoluna limnophila MWH-VicMua1 [39] and Betaproteobacterium *Polynucleobacter necessarius* ssp. *asymbioticus* MWH-Mekk-D6 [40] grown in liquid NSY medium [41] on a rotary shaker at room temperature. Methane-oxidizing bacteria (MOB) type I *Methylomonas methanica* (LW13) and MOB type II *Methylosinus trichosporium* (OB3b) were cultured using nitrate mineral salts medium (NMS; 30 mL) [42] under a methane and air gas phase (50:50 v/v) and incubated at 30°C for 2 days. The purity of these cultures was checked using solid NMS medium supplemented with 10% LB Broth Miller medium (Luria-Bertani, Difco). MOB type I *Methylobacter tundripaludum* SV96^T^ [43] was cultured on M2 medium (Deutsche Sammlung von Mikroorganismen und Zellkulturen, DSMZ; medium 921, pH 6.8, +23°C, 120 rpm) with KNO_3_ as a nitrogen source. The biomass of green sulphur bacterium *Chlorobium phaeobacteroides* DSM 267 (Pfennig 1968, emend. Imhoff 2003) was obtained from the DSMZ (Braunschweig, Germany).

### Terrestrial Carbon Sources

Fallen leaves of dwarf birch (*Betula nana*) from Kilpisjärvi (Finland) were ground to fine particles using a Retch ZM 100 GWB ultra centrifugal mill, and leaves of the common reed (*Phragmites australis*) from Joensuu (Finland) were ground using a Fritsch Planetary Mono Mill Pulverisette. Additionally, we used red alder (*Alnus rubra)* organic matter generated by milling and sieving senesced leaves from Seattle (U.S.A., [13]).

### Stable isotope analyses of bulk biomass

Freeze-dried, homogenized phytoplankton, bacteria and terrestrial matter were weighed (0.6–1.5 mg) in tin cups for δ^13^C analyses, which were carried out on a Carlo-Erba Flash 1112 series Element Analyzer connected to a Thermo Finnigan Delta Plus Advantage Isotope Ratio Mass Spectrometry (IRMS) at the University of Jyväskylä, Finland. Each sample was run in duplicate and compared to the NBS-22 standard using fish muscle and birch as a laboratory-working standard. The precision of the *δ* ^13^C determination (standard deviation of replicate standards) was 0.2‰ for all samples.

### Fatty acid analyses

Lipids were extracted with chloroform:methanol:water (4:2:1) from freeze-dried phytoplankton, bacteria and terrestrial matter samples (1–4 mg). Sonication (10 min) was used to enhance lipid extraction, and samples were centrifuged to facilitate phase separation, after which the chloroform phase was transferred to new tubes. Chloroform was evaporated under a N_2_ gas stream and the remaining lipids were dissolved in toluene. In addition to the total lipid fraction, the phospholipid polar lipid fraction was also obtained via solid phase extraction using silica cartridges (500 mg, Agilent). The cartridges were dehydrated with methanol and preconditioned with chloroform; the sample was introduced in chloroform and then eluted with 10 ml chloroform and 10 ml acetone. The polar lipid fraction was eluted with 10 ml of methanol and evaporated to dryness.

Methanolic H_2_SO_4_ (1% v/v) was added to produce FA methyl esters, and samples were transmethylated in a water bath at 50°C overnight. FA methyl esters were extracted twice with *n*-hexane, and excess *n*-hexane was evaporated under N_2_. The samples were stored at -20°C until analysis.

Fatty acid methyl esters were analyzed using a gas chromatograph (Shimadzu Ultra) equipped with a mass detector (GC-MS) at the University of Jyväskylä (Finland). An Agilent DB-23 column (30 m x 0.25 mm x 0.15 µm) was used with the following temperature program: 60°C for 1.5 min, then the temperature was increased at 10°C min^-1^ to 100°C, followed by 2°C min^-1^ to 140°C, and 1°C min^-1^ to 180°C and finally heated at 2°C min^-1^ to 210°C and held for 6 min. Helium gas was used as a carrier gas with an average velocity of 34 cm sec^-1^. Identification of fatty acids was based on retention times of standards and mass spectra, and we used specific ions as reference ions for identification of each fatty acid methyl ester. The location of the double bond of MUFA (monounsaturated fatty acids) was verified with dimethyl disulphide (DMDS) adducts [44]. For quantification we used a characteristic mass ion ratio (*m/z*) for each fatty acid group. Fatty acid concentrations were calculated using calibration curves based on known standard solutions of a FAME standard mixture. The Pearson correlation coefficient was >0.99 for each individual FA calibration curve. Full description of the method can be found elsewhere [45].

### Stable isotope analyses of total lipids and phospholipids

The total lipid and phospholipid contents of bacteria, phytoplankton and terrestrial matter were measured gravimetrically using the following protocol: after chloroform:methanol extraction, extracts were dried under nitrogen and 500 μL of chloroform was added to vials and 100 μL of each sample with replicateswas transferred to smooth-wall tin capsules. Also, 100 μL replicates of phospholipids were transferred to smooth-wall tin capsules. Capsules were allowed to dry overnight under a fume hood, after which the weight of lipid was measured. The δ^13^C values of the total lipids and phospholipids were then determined with an EA-IRMS system using the method described above. A blank sample of 100 μL of chloroform was used in stable isotope runs to confirm that all chloroform was evaporated from the capsules.

### Gas Chromatography Combustion Stable Isotope Ratio Mass Spectrometry (GC-C-IRMS)

The δ^13^C values of FAs were determined using a GC-C TA III connected to an Isotope Ratio Mass Spectrometer (IRMS, DELTAPLUSXP, Thermo Co.) at the Department of Environmental Sciences of the University of Eastern Finland, Kuopio, Finland. Fatty acids were separated using a 30 m DB-23 column (0.25 mm x 0.15 mm) and then oxidized to carbon dioxide in an oxidation reactor at a temperature of 940°C with the reduction reactor kept at 630°C. The temperature program of the GC column started at 50°C and was kept for 1 minute at 50°C, after which the temperature was raised by 30°C min^-1^ to 140°C, and then by 1°C min^-1^ to 220°C, and finally by 15°C min^-1^ to 300°C. The total run time was 94.3 minutes. The injector temperature was kept at 270°C. The samples were run against an internal standard, >99% hexadecanoic acid methyl ester (C_17_H_34_O_2_, Indiana University, Arndt Schimmelmann), with a δ^13^C value of -30.74‰. This hexadecanoic acid methyl ester standard was used for drift and linear correction. For linear correction, four different concentrations of hexadecanoic were run after which a correction equation was calculated. For the hexadecanoic acid standard the calculated precision was ± 0.6‰ and the accuracy was ± 0.3‰. To take into account possible δ^13^C changes during methylation, precision and accuracy were also calculated using tridecanoic and nonadecanoic acid methyl ester standards, which were first run with an EA-IRMS (DELTAPLUSXP, Thermo Co.) and then calculated for every GC-C-IRMS sample run. The calculated accuracy of these samples was ± 0.9‰, and the precision was ± 0.6‰. Only peaks with a total height of 50 mV at mass 44 were counted. The δ^13^C value of individual FAs was manually calculated using individual background values.

### Data analyses

In total, 67 fatty acids were included in the data set used for detecting differences in FA composition amongst the phytoplankton, bacteria and terrestrial plants. We used permutational multivariate analysis of variance (PERMANOVA; [46]) with unrestricted permutation of raw data and type III sums of squares to test whether these groups had significant differences in their FA composition, and visualized the results in a principal component analysis (PCA) plot.

We also used PERMANOVA to test for differences in the average δ^13^C isotopic difference between FA groups (SAFA, MUFA, ω-3-PUFA, ω-6-PUFA, biomarkers and all fatty acids) and biomass amongst the phytoplankton classes, and visualized the patterns in a non-metric multidimensional scaling (NMS) plot. All of the multivariate analyses operated on euclidean distances of untransformed data. Phytoplankton strains 1, 15 and 19 were excluded from the statistical analyzes of δ^13^C isotopic difference between FA and bulk biomass because of missing data for ω6-FA. NMS is a dimension reduction method that preserves the rank-order of the distances between samples. The stress value is a measure of how well the data can be presented in fewer dimensions (generally 2–3), with values of ca. <0.15 indicating a useful ordination. Samples that are close to each other in the NMS ordination have a similar δ^13^C isotopic difference between the group of fatty acid and bulk tissue, while samples far apart have large differences in their δ^13^C isotopic values.

### Isotopic difference (Δ)

The difference between δ^13^C of lipids/phospholipids/fatty acid and δ^13^C of bulk biomass s was calculated using the following equation [47,48]:

$$\delta^{13}C_{lipod/PL/biomar\ker FA-biom}=\delta^{13}C_{lipid/PL/FA/biomar\ker FA-}\delta^{13}C_{biom}$$ (2)

where δ^13^C_biom_ is the δ^13^C value of the bulk biomass, δ^13^C_lipid_ is the δ^13^C value of total lipid fraction, δ^13^C_PL_ is the δ^13^C value of the phospholipid fraction, ^13^C_FA_ is the δ^13^C value of the individual fatty acids, and δ^13^C_biomarker FA_ is the average of δ^13^C values of the group characteristic fatty acids, called here as biomarkers (biomarker FA) (Table 2). The term isotopic difference (Δ) is used here when discussing the distributions of isotopes between substances.

**Table 2:** Isotopic fraction between lipids and bulk biomass of phytoplankton, terrestrial matter and bacteria. The biomarker fatty acids (FA) used for δ^13^C analysis were characteristic of each strain. Carbon, lipid and phospholipid (PL) content of selected groups of phytoplankton, bacteria and terrestrial organic matter, are averages of selected strains (see Table 1). Isotopic difference (Δ) were calculation by subtracting the δ^13^C value of lipid, phospholipid (PL) or biomarker fatty acid (biomarker FA) from the δ^13^C value of bulk biomass (biom).

Functional group	FA δ^13^C biomarker	Carbon content (% of DW)	Lipid content (% of DW)	PL content (% of DW)	Δ δ^13^C_lipid-biom_	Δ δ^13^C_PL-biom_	Δ δ^13^C_biomarker FA-biom_
**Phytoplankton group**							
*Chlorophyceae*	16:4ω3	52.4±1.5	18.9±1.9	3.3±0.8	-4.2±1.8	-5.1±2.4	-9.9±1.2
*Cryptophyceae*	18:4ω3, 22:5ω6	50.7±1.0	10.4±1.4	2.3±0.2	-5.1±1.0	-5.6±0.5	-7.0±0.7
*Chrysophyceae*	18:4ω3, 22:5ω6	44.2±0.6	10.9±1.6	nd	-5.0±0.4	nd	-7.1±0.9
*Cyanophyceae*	16:1ω7*, 18:3ω6, 20:3ω6	47.5±4.7	6.6±1.7	1.2±0.9	-5.5±0.9	-6.3±1.3	-11.4±0.65
*Bacillariophyceae*	16:2ω7, 16:2ω4, 16:3ω4	34.1±1.5	8.8±1.9	1.0±0.1	-4.2±0.1	-5.0±0.1	-4.1±0.83
*Dinophyceae*	18:2ω6, 18:5ω3	49±3.8	8.6±3.7	1.8±0.1	-1.9±2.1	-0.8±1.8	nd
*Euglenophyceae*	20:2ω6, 20:3ω6, 20:3ω3				nd	nd	-6.0±0.5
*Rahpidophyceae*	16:2ω4				nd	nd	-6.3±0.28
**Microbial group**							
**Heterotrophic bacteria**							
*Gram-*	Cy-19:0t	47.5±0.1	6.8±1.8	3.9±0.1	0.4±0.1	0.6±0.0	-0.4±0.6
*Gram+*	i-14:0, a-15:0, i-15:0	45.8±0.4	7.9±2.0	4.1±0.8	-2.0±0.1	-0.7±0.6	-2.2±0.1
**Methanotrophic bacteria**							
*Type 1*	16:1ω6c, 16:1ω5t	37.3±8.5	nd	nd	nd	nd	-8.0±4.4
*Type 2*	18:1ω6c, 18:1ω5c	42.1±0.0	nd	nd	nd	nd	-3.4±1.4
**Green sulphur Bacteria**							
*Chlorobium*	15:0, 15:1ω7	26.2±1	nd	nd	nd	nd	-4.7±0.2
**Terrestrial OM**							
*Betula*	20:0, 22:0, 23:0, 24:0	48±0.5	5.7±1.3	0.7±0.1	-3.0±0.1	-2.9±0.2	nd
*Phragmites*	20:0, 22:0, 23:0, 24:0	29.6±0.6	1.7±0.1	0.1±0.0	-6.0±0.1	-7.5±0.2	-6.6±1.2
*Alnus*	20:0, 22:0, 23:0, 24:0	46.1±1.6	3.9±0.9	0.4±0.0	-2.7±0.1	-1.9±0.2	nd

## Results

### Carbon and lipid content

The total carbon content of dry weight (DW) was similar (45.2±7.6% of DW) amongst the phytoplankton, bacteria and terrestrial matter samples with only a few exceptions (Table 2). Amongst the phytoplankton, the carbon content was highest in Chlorophyceae (52.4±1.5%) and lowest in Bacillariophyceae (34.1±1.5%). Heterotrophic bacteria (46.7±1.2%) and terrestrial tree leaves (46.1±1.6%) had similar carbon content compared to phytoplankton, but lower carbon content was found for the grass *Phragmites* (29.6±0.6%) and the bacterium *Chlorobium* (28±2.1%). The lipid content was higher amongst the phytoplankton (9.5±4.0%) than bacteria (7.3±0.8%) or terrestrial plants (3.9±1.7%) (Table 2). However, the total lipid content varied between the studied organisms being highest in Chlorophyceae (18.9±1.9%) and lowest in *Phragmites* (1.7±0.1% of DW). The average phospholipid content was only 1.9±1.3% of DW amongst all phytoplankton, bacteria and terrestrial matter, but was slightly higher in phytoplankton (2.1±1%) and in bacteria (4.0±0.1%) than in terrestrial matter (0.4±0.3%).

### Fatty acid biomarkers

**Fig 1:**
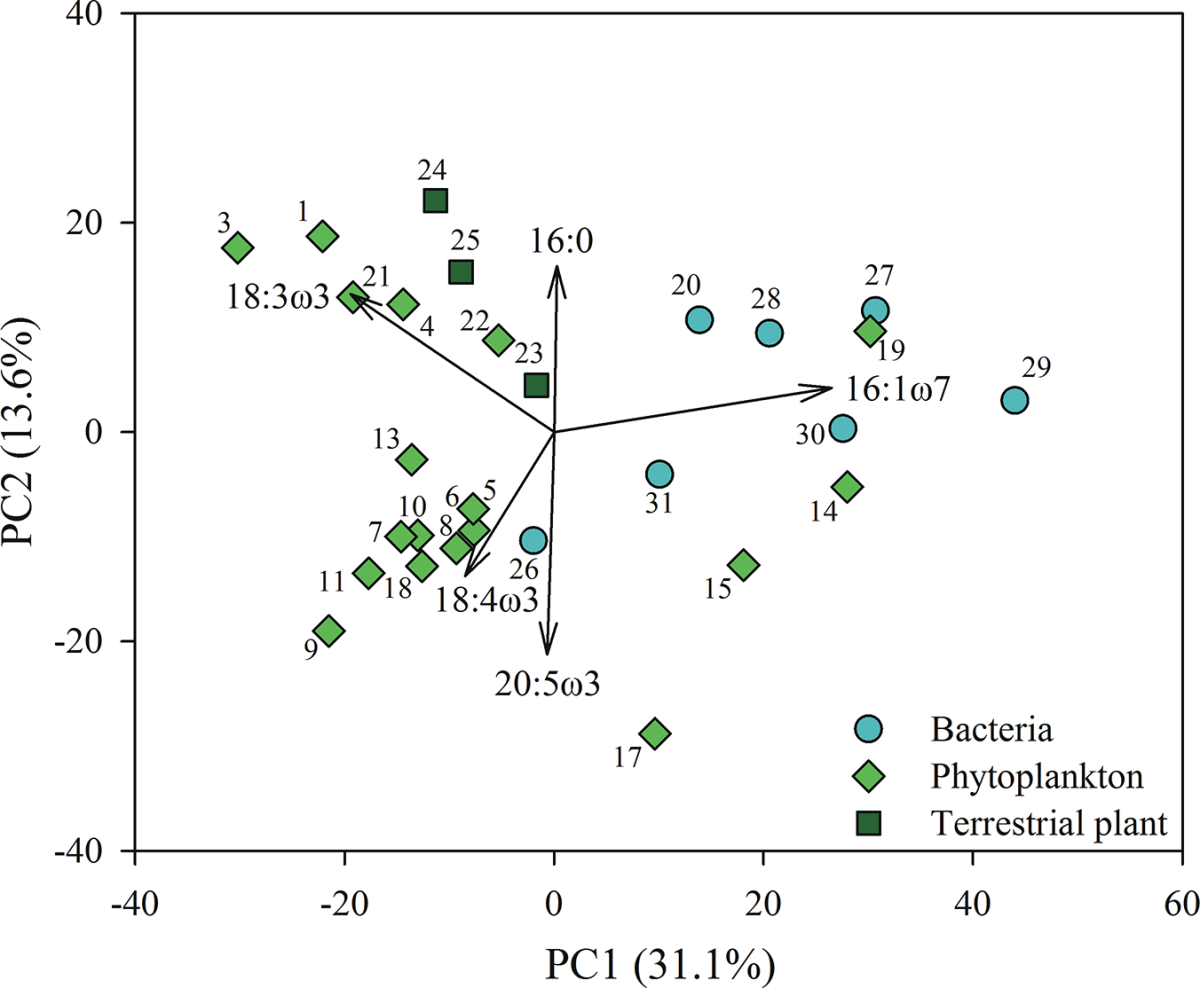
Fatty acid profiles of phytoplankton, bacteria and terrestrial plants. Principal component analysis (PCA) of the fatty acid composition of the phytoplankton, bacteria and terrestrial plants. Proportion of explained variance is in parentheses. PCA was run with all 67 fatty acids, but only eigenvectors > 0.3 are shown.

The FA profiles of the phytoplankton, bacteria and terrestrial matter differed greatly (S1 Table). In total, 67 different FAs were determined from the phytoplankton, bacteria, and terrestrial plants, many of which were characteristic for the different groups. The FA composition of phytoplankton, bacteria and terrestrial OM differed significantly (Fig 1, PERMANOVA, F_2,25_ = 3.680, p < 0.001; pairwise comparisons: *t* = 1.523–2.202, *p* < 0.03); and these differences can also be seen in the PCA, although the two axes captured a relatively low proportion of the total variance (44.7%), with diatoms and bacteria clustered on the right side of the PCA plot, and other phytoplankton and terrestrial plants clustered on the left side. From all the FA quantified, we selected characteristic FAs, which were not prevalent or even found in other groups, as specific biomarker FA (Table 2). Other criteria for these biomarkers were high concentrations and good precision between compound-specific runs. Therefore, some of the biomarkers selected for compound specific runs differed from traditional biomarkers in marine or freshwater studies. All of the phytoplankton biomarkers selected belonged to the ω-3 or ω-6 series and thus were clearly distinct from those of bacteria and terrestrial matter. Distinctions between phytoplankton were made based on the C_16_, C_18_, C_20_ and C_22_ polyunsaturated FA (PUFA).

*Iso*- and *anteiso-*branched FAs were used as biomarkers for heterotrophic *Candidatus* Rhodoluna limnophila (Actinobacteria; Gram+), whereas cy-19:0t was used as a specific FA biomarker for *Polynucleobacter necessarius* ssp. *asymbioticus* (Betaproteobacteria; Gram-). For autotrophic green sulphur bacteria (phylum Chlorobi, *Chlorobium* sp.) we used the FA 15:0 and 15:1ω7c_._ The FA 16:1ω8c, 16:1ω6c and 16:1ω5t monounsaturated FA characterized the MOB type I (Gammaproteobacteria), but due to the co-elution of 16:1ω7c and 16:1ω8c only 16:1ω6c and 16:1ω5t were used to estimate the δ^13^C value of MOB. Similarly, 18:1ω6c and 18:1ω5t were used as specific biomarkers for MOB type II (Alphaproteobacteria). Furthermore, terrestrial plants could be separated from phytoplankton and bacteria through their high content of long chain saturated FAs (C_20_-C_30_), which are ideal biomarkers for terrestrial plants.

### The δ^13^C values of bulk biomass and fatty acids

The bulk δ^13^C values of the phytoplankton, bacteria and terrestrial matter and the δ^13^C values of the individual FA are presented in S2 table. The δ^13^C values of bulk biomass differed widely between organisms. The most depleted δ^13^C value (-69.1±0.1‰) was measured from type II MOB (*Methylosinus trichosporium*) and the least depleted δ^13^C value (-13.6±0.6‰) was measured from the Chlorophyte *Scenedesmus gracilis*. The δ^13^C values of the phytoplankton showed some differences between different cultures and between taxa grown under the similar culture conditions (S2 Table); however, this topic is not the focus of this study and these results were not pursued further.

The δ^13^C values of all FA within the phytoplankton, bacteria and terrestrial matter varied within each strain with SD ranging from 0.9 to 5.8‰. The δ^13^C values of all FA were most consistent (SD<2.2‰) amongst the Chlorophyceae, Chrysophycea, *Chlorobium* and heterotrophic bacteria, and least consistent (SD = 4.1–5.8‰) in *Euglena*, *Phragmites*, *Methylobacter* and *Methylosinus*. The high deviation in *Euglena* was related to the number of carbons in the FA, the most depleted FA having 22 carbon molecules in the FA chain. Amongst all FA of the cultured MOBs, the δ^13^C values of 16:1ω8c or/and 18:1ω8c were higher and 16:1ω6c/5t lower in relation to other FA. The δ^13^C values of biomarker FA were on average more consistent than other FA and SD values were was less than ±2‰ for all of the studied phytoplankton, bacteria and terrestrial matter samples.

### Isotopic difference of lipids and fatty acids

**Fig 2:**
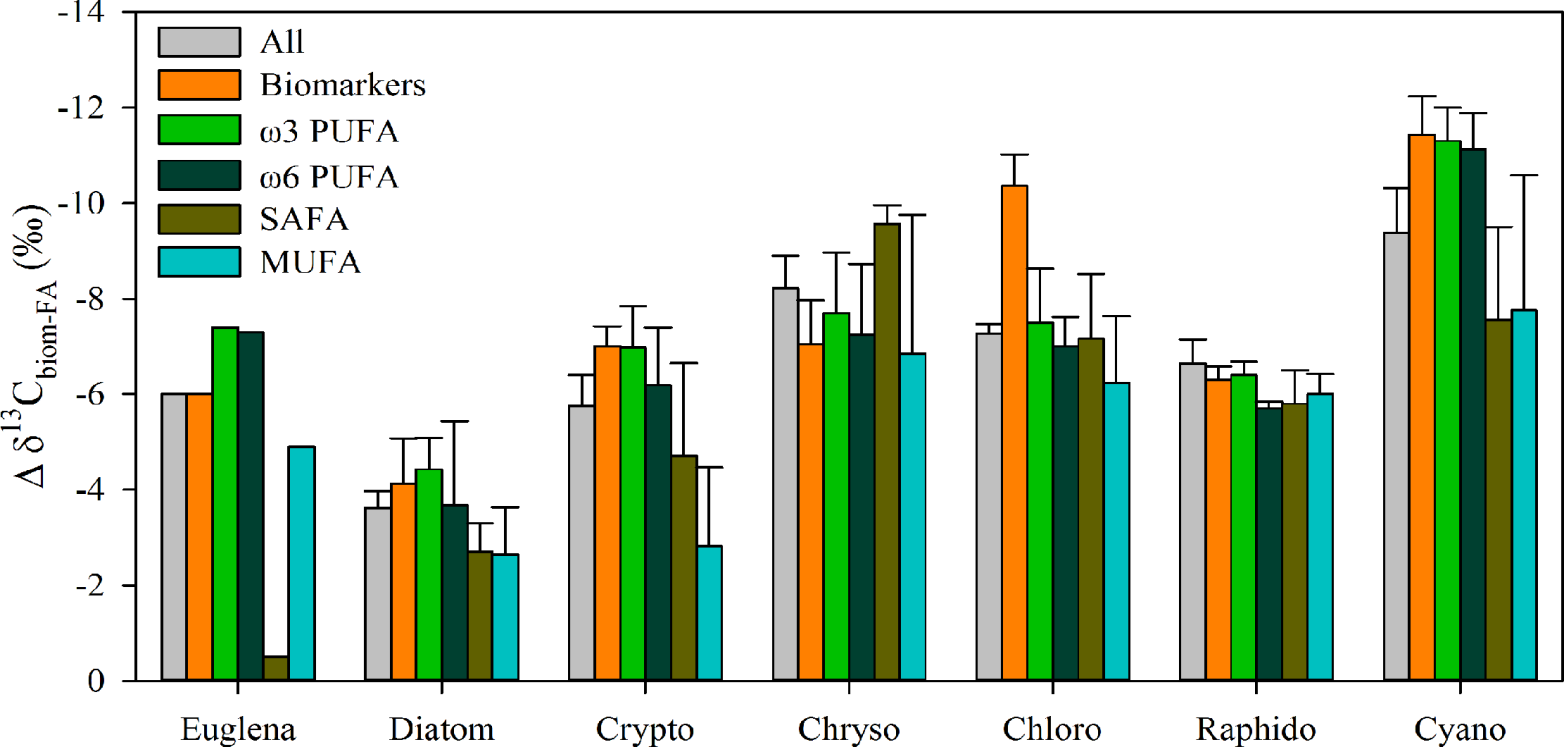
Isotopic difference between fatty acid and bulk biomass of freshwater phytoplankton. The carbon isotopic difference (Δ δ^13^C, mean ± SD) between fatty acid groups and bulk biomass varied amongst the phytoplankton classes.

**Fig 3:**
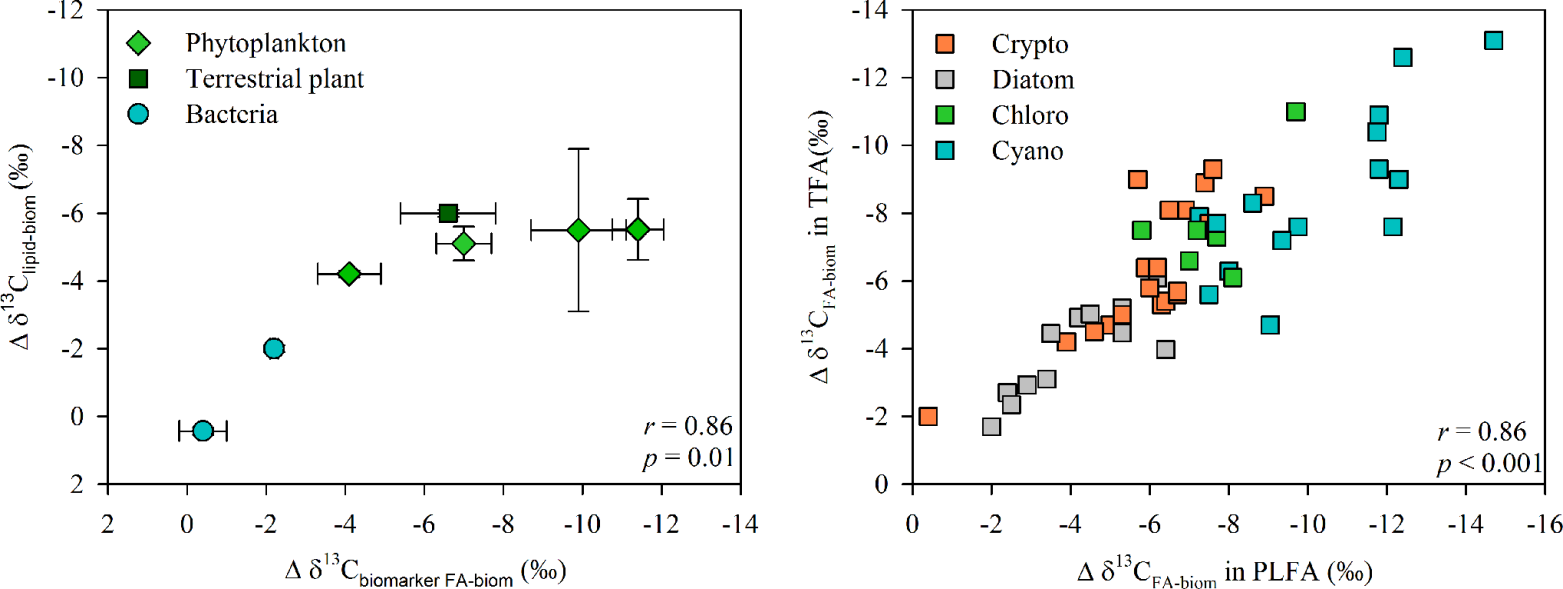
Isotopic difference between lipids or fatty acids and bulk biomass. a) The carbon isotopic difference (Δ δ^13^C) between total lipids and bulk biomass is strongly correlated with the isotopic difference between biomarker fatty acids and bulk biomass for phytoplankton, bacteria and terrestrial organic matter. b) Carbon isotopic difference between total fatty acids and bulk biomass is strongly correlated with that between bulk biomass phospholipid fatty acids.

Carbon (δ^13^C) isotopic difference between lipid/phospholipid FA and bulk biomass of the cultured phytoplankton, bacteria and terrestrial matter are presented in Table 2, and for phytoplankton alone in Fig 2. Total lipid and phospholipid extractions of phytoplankton, bacteria and terrestrial matter were similarly depleted or enriched in ^13^C relative to bulk biomass (Pearson’s correlation r = 0.967, p<0.01). Additionally, the carbon isotopic differences between lipids and bulk biomass were strongly correlated with the carbon isotopic differences between FA and bulk biomass (Pearson’s correlation r>0.89, p<0.01, Fig 3). The carbon isotopic difference between total lipids or phospholipids and bulk biomass was similar (-4.8±0.6‰ and -5.5±0.6‰, respectively) amongst all phytoplankton classes except Dinophyceae. The carbon isotopic difference between total lipid or phospholipid fraction and bulk biomass was less pronounced in *Alnus* and *Betula* than in *Phragmites*. The carbon isotopic difference between lipids and bulk biomass was close to zero in Betaproteobacteria but was -1.4±0.9‰ in Actinobacteria.

**Fig 4:**
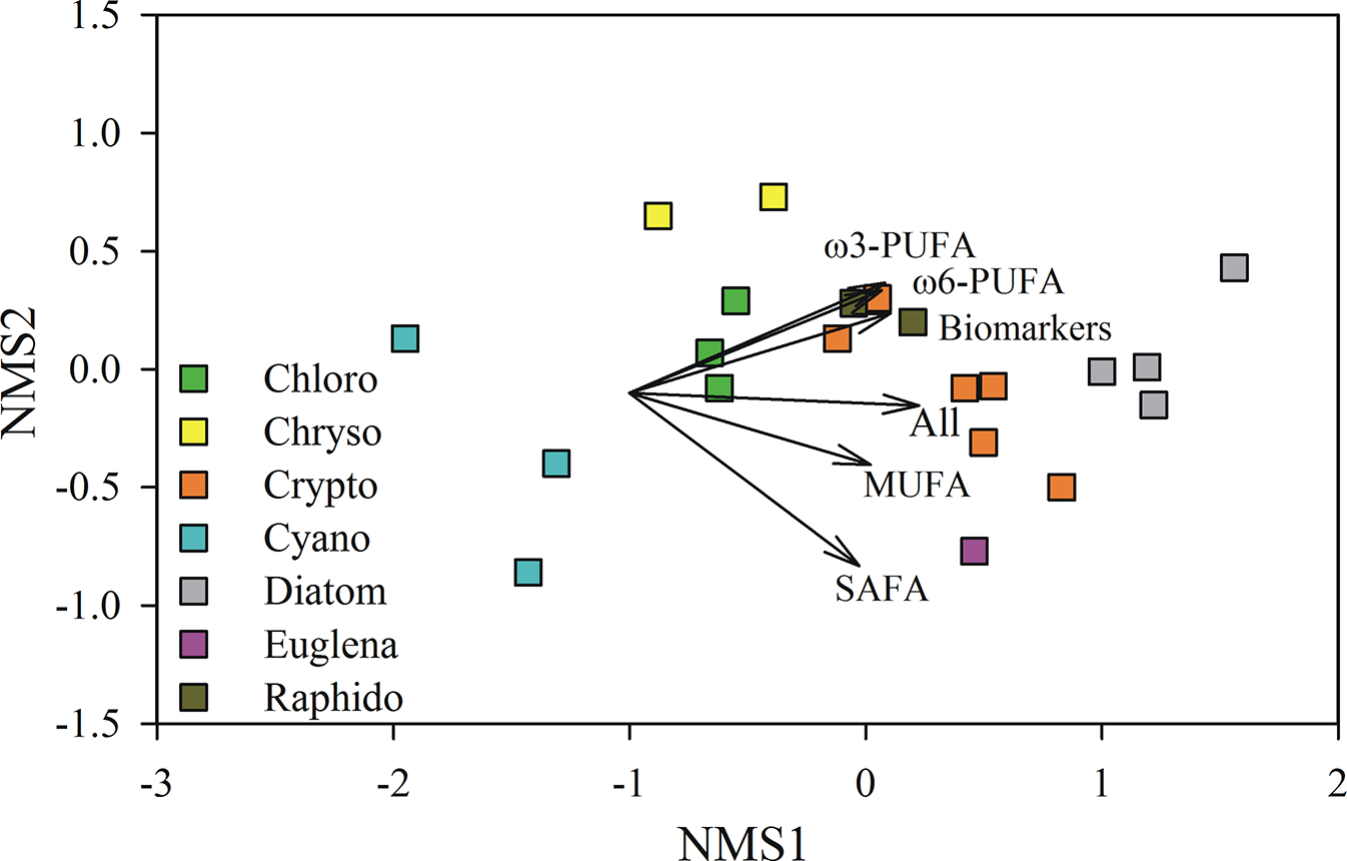
Isotopic distinction between freshwater phytoplankton by their δ^13^C value of fatty acids. A non-metric multidimensional scaling (NMS) plot visualizing the amongst-class differences in the phytoplankton isotopic difference (Δ δ^13^C) between the bulk biomass and fatty acid groups. Stress for the 2-dimensional solution was 0.06, and the variables are presented as vectors.

Class identity explained 78% of the variation in the phytoplankton carbon isotopic difference between FA and the bulk biomass and, and this differed significantly amongst most of the 7 phytoplankton classes (Fig 4, PERMANOVA, F_6,14_ = 11.281, p < 0.001). Total lipid fatty acids and the phospholipid fatty acids of phytoplankton, bacteria and terrestrial matter were similarly depleted or enriched in ^13^C relative to average biomass, except for Cyanobacteria, which were systematically more ^13^C depleted in the PLFA fraction than in the total fraction (S2 Table). The carbon isotopic difference between FA groups and bulk phytoplankton biomass varied amongst the phytoplankton classes, but also amongst the different FA groups (Fig 2, Fig 4). The carbon isotopic difference between biomass and FA groups was most stable in *Gonyostomum semen* (Raphidophyte), and varied the most in *Euglena gracilis*. However, the carbon isotopic difference between PUFA and bulk phytoplankton biomass had less uncertainty than amongst the other FA groups (e.g., SAFA), apart from the chlorophytes for which the biomarker FA (16:4ω3) was more ^13^C-depleted than other PUFA in relation to the average biomass. Therefore, the δ^13^C value of phytoplankton within the seston could be calculated using the δ^13^C value of any PUFA. This is advantageous because biomarker PUFA make it possible to distinguish different co-occurring phytoplankton.

The carbon isotopic difference between all FAs or biomarker FAs and bulk biomass, amongst Chlorophyceae (*Selenastrum*, *Scenedesmus*, *Chlamydomonas*) was -7.0±1.7‰ and -9.9±1.2‰, respectively, with little difference amongst the different strains tested. Amongst the Cryptophyceae (*Cryptomonas*, *Rhodomonas*), the carbon isotopic difference between all FA, or biomarker FA and bulk biomass was similar across strains with an average of -5.6±1.9‰ and -7.0±0.7‰, respectively. Additionally, the carbon isotopic difference between biomarker FA and bulk biomass was similar between Cryptophyceae and Chrysophyceae (-7.1±0.9‰). However, the carbon isotopic difference between all FA and bulk biomass was greater among Chrysophyceae (-7.8±1.5‰) than Cryptophyceae (-5.9±2.2‰). In the Cyanobacteria, different biomarker FAs were used for *Microcystis* and *Synechococcus*, as *Synechococcus* does not contain 18:3ω6, but had a high proportion of 16:1ω7, which is also a common FA in many gram-negative bacteria and diatoms and thus cannot be used as a specific biomarker for *Synechococcus*. The carbon isotopic difference between selected biomarker FA and bulk biomass was similar (-11.4±0.7‰) for the two different *Microcystis* cultures and *Synechococcus*. Nevertheless there was more variation (SD = ±3.7‰) in the isotopic difference between the monounsaturated FAs and bulk biomass for the cyanobacteria strains. Bacillariophyceae (*Fragillaria*, *Cyclotella*) had the lowest isotopic difference amongst all of the phytoplankton between biomarker FA (-4.1±0.8‰) or all FA (-4.0±1.7‰) and bulk biomass. There was considerable variation in the carbon isotopic difference between FAs (-6.0±4.4‰) and bulk biomass of *Euglena gracilis*, although variation was less amongst selected biomarker FA (-6.0±0.5‰).

Amongst the heterotrophic bacteria, the carbon isotopic difference between the biomarker FA and bulk biomass was -2.2±0.1‰ for Actinobacteria, -0.4±0.6‰ for Betaproteobacteria, and -4.7±0.2‰ for *Chlorobium*. The carbon isotopic difference between biomarker FA and bulk biomass was similar for *Methylobacter* and *Methylomonas* with an average of -8.0±4.4‰, but was only -3.4±1.4‰ for *Methylosinus* type II MOB.

## Discussion

In the virtual absence of direct δ^13^C values for phytoplankton, bacteria and terrestrial matter from mixed seston, different indirect methods have been developed to estimate these values. These indirect methods have either been based on the use of substrates and assumed fractionation factors or have tried to track and separate distinct sources from seston using chlorophyll-*a* content or nitrogen and hydrogen isotopes with mixing models [17, 49]. However, all these methods have serious shortcomings [17]. For example, when using the lake DIC δ^13^C value with an assumed photosynthesis fractionation factor to estimate phytoplankton δ^13^C, there is high uncertainty in the bulk δ^13^C estimate due to the fact that DIC δ^13^C in lake water can vary widely [18, 19], while the photosynthesis fractionation can also vary between 0 and -20‰ [15, 18]. Biomolecules offer a semi-direct method for determining bulk phytoplankton, bacteria and terrestrial matter δ^13^C values because the biomolecules originate from the organisms themselves and thus only the isotopic carbon difference is required to convert δ^13^C for the biomolecule to bulk cell δ^13^C values. Here we used lipids and FAs as specific biomarkers to overcome the shortcomings of existing indirect methods. Our results show the carbon isotopic carbon difference between lipids and bulk tissue to be rather consistent amongst algae and bacteria at the class level. Our semi-direct method has a precision (SD in δ^13^C values of biomarker FA) of ≈±1.4‰ for the carbon isotopic difference between biomarker FAs and bulk biomass for phytoplankton, bacteria (excluding MOB type I), and terrestrial matter, which would also be the maximum uncertainty of calculated δ^13^C values for these specific resources. This uncertainty includes analytical bias and also the impact of culture conditions on the carbon isotopic difference. This semi-direct method is a substantial improvement over earlier methods.

Fatty acids have been previously shown to be effective taxonomic biomarkers for freshwater phytoplankton and marine macrophytes [30, 45, 50]. Additionally, a recent study of Galloway and Winder [51] showed that various environmental factors including nutrients, temperature, and light also impact FA profiles. However, these authors also found taxonomic affiliation explained 3–4 times more FA variation than did environmental conditions. Futhermore, during summer stratification the surface temperature of boreal and temperate lakes is usually between 15°C and 25°C, and even a temperature increase from 25 to 35°C had a relatively small impact on the abundance of ω-3 in Australian microalgae [52], thus our results should be applicable for lakes in different climate zones.

We selected specific FAs for each taxon so that the δ^13^C of FAs approach could separate phytoplankton, bacteria and terrestrial matter from each other at the taxa level in mixed seston samples. Our examination of 8 phytoplankton classes, 5 non-phototrophic bacterial taxa and 3 terrestrial plants revealed that δ^13^C difference between the FA and the bulk biomass of the studied phytoplankton, bacteria and terrestrial plants follows taxonomic categories. Our results also show a strong correlation between the isotopic difference of total lipids/phospholipids/fatty acids and bulk biomass on tested cultured conditions. Therefore, total lipid extractions from seston can be used to derive δ^13^C values for phytoplankton and bacteria. In phytoplankton, the carbon isotopic difference between the average of all FAs and the bulk biomass appears to be rather stable within the taxa we studied (Table 2). Additionally, our biomarker FA approach turned out to be more stable and reliable than the average carbon isotopic differences for all FAs. In the case of bacteria, the carbon isotopic difference between the average of all FA and bulk biomass was low for Actinobacteria and Betaproteobacteria, but higher for MOB and autotrophic green sulphur bacteria.

The calculated isotopic differences between biomarker FAs and bulk biomass were systematically similar amongst the phytoplankton species within the same class (SD ≈±1.2‰), but varied between classes showing the importance of differentiating phytoplankton at the class level when estimating the δ^13^C value of phytoplankton in ecological studies. Differences between classes might indicate different biosynthetic pathways and enzymatic processes. The acetogenic pathway is used for alkyl lipids and n-fatty acids, whereas the mevalonic-acid pathway is used for isoprenoid lipids (sterols and hopanoids) and the methylerythritol-phophate pathway for isoprenoid lipids (phytols and hopanoids in cyanobacteria) [38]. In cells that contain a chloroplast, the chloroplast is usually the site for fatty acid synthesis. However, in cells without chloropasts FA are produced in the cytosol [36]. According to the theoretical calculations of Hayes [36], a decrease in abundance of lipids relative to carbohydrates may increase the isotopic difference between biomass and lipids, and thus increase isotopic depletion (e.g., from -2‰ to -4‰). However, the impact of cellular composition is estimated to be less than ±1‰ in marine systems, which is less than the uncertainty for our method. Additionally, the lipid content of our cultured phytoplankton or bacteria did not correlate with the lipid-biomass isotopic difference.

The clear in lipid isotopic difference between Bacillariophyceae and the other eukaryotes, emphasizes that each phytoplankton group should have its own lipid isotopic difference value as well as its own characteristic PLFA. Additionally, even though we were not able to run δ^13^C from the FA of *Peridinium* (Dinophyceae), we found that in this dinoflagellate the carbon isotopic difference between total lipids or phospholipids and the bulk biomass is much lower than any other phytoplankton class. The reason for this is not known, but raises the question of whether mixotrophy could affect the carbon isotopic difference, as many dinoflagellates, including *Peridinium*, are known to be mixotrophic [53]. Nevertheless, even though our cultures were not axenic and contained some bacteria there was little variation between species. Even if our culture conditions differed from previous studies [36, 37] our FA isotopic difference factor (-7.0±0.7‰) for Cryptophytes was similar to those from previous studies. Differences between cyanobacteria and other phytoplankton groups may reflect the fact that the former are prokaryotic and the latter eukaryotic. However, the values for cyanobacteria also clearly differed from those for other autotrophs as well as heterotrophic bacteria.

For some organisms (e.g., Actinobacteria) the δ^13^C values for different FAs were very consistent (SD<±1‰). In general, variation (SD) was usually ≈ ±2.2‰ excluding *Euglena*, *Phragmites*, *Microcystis*, *Synechococcus* and all MOB cultures (SD< ±4.6‰). Generally it was noted that the δ^13^C values of the FA groups were least stable amongst the SAFA, which usually have more enriched δ^13^C values and thus should not be used to estimate δ^13^C of bulk biomass. The more ^13^C-enriched values of SAFAs can possibly be explained by the fact that excess carbohydrates are converted to palmitic acid (16:0) [54] and then further elongated to stearic acid (18:0). Therefore the δ^13^C value of the saturated FA is also impacted by the carbohydrates, which are generally more ^13^C-enriched than FA [37]. Furthermore, the most common SAFAs (i.e., 16:0 and 18:0) are ubiquitous in algae, terrestrial plants and bacteria and therefore have no utility as biomarkers. We also found that the δ^13^C values of long chain PUFAs were more ^13^C-depleted than other FAs, which might result from the elongation or desaturation processes during the synthesis of these FAs.

Our results for the heterotrophic Actinobacteria and Betaproteoabacteria showed a -0.4 and -2.0‰ carbon isotopic difference between FAs and bulk biomass, respectively. These results are close to the previous measurements by Blair [55], Hayes [56] and Cowie et al. [57] who reported -2 to -3‰ lipid fractionation in heterotrophic bacteria. Our repeated measurements of gram positive Actinobacteria showed low variation in the δ^13^C values of FAs, which simplifies their recognition in pelagic samples [58]. The calculation of δ^13^C for heterotrophic gram negative bacteria is more difficult due to the lack of a specific bacterial biomarker, except in our study organism *Polynucleobacter* sp. which contains cyclo-19:0 which had isotopic difference value of -0.4±0.6‰.

Those MOB that use the ribulose monophosphate pathway, e.g. *Methylomonas*, *Methylomicrobium*, *Methylobacter*, *and Methylococcus*, are reported to have a similar δ^13^C isotopic difference (-2 to -6‰) as heterotrophic bacteria [33,56]. However, in our study this was true for *Methylomonas methanica*, but not for the slower growing *Methylobacter tundripaludum* which had a higher isotopic difference of -10 to -12‰. Altogether our results suggested a -8.0±4.4‰ isotopic difference between biomarker FAs and bulk biomass for MOB type I. This 4‰ uncertainty is still a large improvement over earlier methods which estimated the δ^13^C of MOB biomass using the fractionation between the carbon source and biomass (-7.8 to -28.4‰; [24]). Interestingly, our low isotopic difference (~3‰) for *Methylosinus trichosporium* contrasts with earlier studies of MOB type II which proposed a high fractionation between bulk biomass and FAs because of the serine pathway [33, 59].

We conclude that compound specific analyses of biomarker FAs offer a promising tool for more precise δ^13^C determinations for the phytoplankton, bacteria and terrestrial organic matter embedded within lake seston. Even more accurate results can be obtained through δ^13^C estimation from specific biomarker FA. The reproducibility of δ^13^C isotopic difference is high for phytoplankton and thus the analyses can and should be made at least at the class level. Similarly, the biomarker FA together with the δ^13^C isotopic difference can be used for heterotrophic gram positive bacteria, type I and type II MOB and green sulphur bacteria. When compound-specific stable isotope analyses of specific biomarkers becomes more widely available and cheaper, it may finally become possible to routinely separate phytoplankton and bacteria from terrestrial matter and other components of the seston. Such analyses could elevate aquatic food web studies to a much higher level of resolution.

## Conclusion

Stable isotopes are a useful tool for determining consumer diets and this approach has recently been used to quantify allochthonous and autochthonous subsidies in zooplankton diets [1–6]. Whereas diet stable isotope values can be easily determined in laboratory experiments, various indirect methods have been used in field studies resulting in great uncertainty in results [17]. Here we established a semi-direct compound specific method for deriving bulk δ^13^C values for phytoplankton, terrestrial matter and bacteria by determining lipid and fatty acid δ^13^C values and calculating the isotopic difference between lipids/fatty acids and bulk biomass. Our results showed that when using the δ^13^C value of fatty acids, the bulk biomass of phytoplankton and bacteria (excluding MOB) can be calculated with ±1.4‰ precision.

## Supporting Information

S1 Table Fatty acid profiles of freshwater phytoplankton, bacteria and terrestrial matter. Percentages (%, average) of saturated fatty acids, branched fatty acids, monounsaturated fatty acids and polyunsaturated fatty acids of studied freshwater phytoplankton, bacteria and terrestrial plants. Sum of each group from total contribution is also represented.
(PDF)

S2 Table The δ^13^C value of fatty acids of studied freshwater phytoplankton, bacteria and terrestrial matter. The δ^13^C value (mean±SD) of saturated fatty acids, branched fatty acids, monounsaturated fatty acids and polyunsaturated fatty acids of studied freshwater phytoplankton, bacteria and terrestrial plants. Average δ^13^C value of each fatty acid group (x±SD) is also represented.
(PDF)
